# Reduced proliferation of endothelial colony-forming cells in unprovoked venous thromboembolic disease as a consequence of endothelial dysfunction

**DOI:** 10.1371/journal.pone.0183827

**Published:** 2017-09-14

**Authors:** Rubicel Hernandez-Lopez, Antonieta Chavez-Gonzalez, Patricia Torres-Barrera, Dafne Moreno-Lorenzana, Norma Lopez-DiazGuerrero, David Santiago-German, Irma Isordia-Salas, David Smadja, Mervin C. Yoder, Abraham Majluf-Cruz, J. Antonio Alvarado-Moreno

**Affiliations:** 1 Unidad de Investigacion Medica en Trombosis, Hemostasia y Aterogenesis, Instituto Mexicano del Seguro Social, Mexico City, Mexico; 2 Posgrado en Biologia Experimental, Universidad Autonoma Metropolitana, Iztapalapa. Mexico City, Mexico; 3 Unidad de Investigacion Medica en Enfermedades Oncologicas, Instituto Mexicano del Seguro Social, Mexico City, Mexico; 4 Servicio de Urgencias, Instituto Mexicano del Seguro Social Mexico City, México; 5 Paris Descartes University, INSERM UMR-S 1140, Faculté de Pharmacie de Paris, Paris, France; 6 AP-HP, Hôpital Européen Georges Pompidou, Hematology department, Paris, France; 7 Wells Center for Pediatric Research, Indiana University School of Medicine, Indianapolis, Indiana, United States of America; Centro Cardiologico Monzino, ITALY

## Abstract

**Background:**

Venous thromboembolic disease (VTD) is a public health problem. We recently reported that endothelial colony-forming cells (ECFCs) derived from endothelial cells (EC) (ECFC-ECs) from patients with VTD have a dysfunctional state. For this study, we proposed that a dysfunctional status of these cells generates a reduction of its proliferative ability, which is also associated with senescence and reactive oxygen species (ROS).

**Methods and results:**

Human mononuclear cells (MNCs) were obtained from peripheral blood from 40 healthy human volunteers (controls) and 50 patients with VTD matched by age (20−50 years) and sex to obtain ECFCs. We assayed their proliferative ability with plasma of patients and controls and supernatants of cultures from ECFC-ECs, senescence-associated β-galactosidase (SA-β-gal), ROS, and expression of ephrin-B2/Eph-B4 receptor. Compared with cells from controls, cells from VTD patients showed an 8-fold increase of ECFCs that emerged 1 week earlier, reduced proliferation at long term (39%) and, in passages 4 and 10, a highly senescent rate (30±1.05% vs. 91.3±15.07%, respectively) with an increase of ROS and impaired expression of *ephrin-B2/Eph-4* genes. Proliferation potential of cells from VTD patients was reduced in endothelial medium [1.4±0.22 doubling population (DP)], control plasma (1.18±0.31 DP), or plasma from VTD patients (1.65±0.27 DP).

**Conclusions:**

As compared with controls, ECFC-ECs from individuals with VTD have higher oxidative stress, proliferation stress, cellular senescence, and low proliferative potential. These findings suggest that patients with a history of VTD are ECFC-ECs dysfunctional that could be associated to permanent risk for new thrombotic events.

## Introduction

The World Health Organization (WHO) reported that thrombosis is the first cause of death and, specifically, contemplates that venous thromboembolic disease (VTD) must be considered a public health problem [1]. VTD is a consequence of several risk factors including family history, genetics, and environment. Searching for new risk factors for VTD may help to better explain the pathophysiology of this disease. We previously hypothesized that it was likely that some unknown factors may reduce the physiological process of vascular regeneration by endothelial progenitor cells (EPCs) [2], in particular endothelial-colony forming cells (ECFCs). EPCs have a robust proliferative potential identified in adult humans and peripheral blood and have the ability of vessel formation *in vivo* [3]. There is evidence that they are present in the circulation of patients with acute myocardial infarction [4] and splanchnic vein thrombosis [5]. These data suggest the need for endothelial precursors in order to repair a dysfunctional vessel wall [6]. Moreover, some proteins such as the EphrinB2-Eph4 complex are required either for revascularization or to begin the cellular responses to initiate regeneration and revascularization of arterial or venous vessels [7]. Some factors may change the normal physiology of EPCs, resulting in different pathological entities. Studies show that high levels of reactive oxygen species (ROS) are involved in chronic human diseases such as obesity, type 2 diabetes, atherosclerosis, and cardiovascular diseases and that they may provoke endothelial dysfunction [8]. We recently reported morphological abnormalities of the mitochondria of ECFC-ECs from VTD patients [9], suggesting mitochondrial dysfunction [10], a mechanism that may link ROS, endothelial dysfunction in ECFC-ECs, increased production of inflammatory cytokines with apoptosis, senescence, and other vascular abnormalities in patients with VTD [11–13]. These events may all induce a reduced cell proliferation. In this study, we examine the effects of several types of cell supernatants and ROS on senescence, apoptosis, and cellular proliferation in ECFC-ECs from VTD patients.

## Materials and methods

### Peripheral blood samples from controls and patients with VTD

One hundred ml of peripheral blood was collected in tubes containing 1,000 IU of a sodium heparin solution (Tecnofarma, México) from 40 healthy human volunteers (controls) and 50 VTD patients matched by age (20−50 years) and gender. Fifteen ml of peripheral blood was collected separately and then centrifuged at 1,000 g for 15 min at room temperature to obtain control and patient plasma. Plasma aliquots were prepared and stored at -70°C for further studies.

General characteristics of VTD patients and controls are shown in Table 1. All patients had a history of recurrent unprovoked VTD (>3 episodes) and were under anticoagulation therapy with vitamin K antagonists (86% of the patients) or with rivaroxaban (14% of the patients). Only 36% of the patients had positive results to be considered as carriers of known thrombophilia. In order to participate in the study, a negative D-dimer test before entering the study was required. The last thrombotic episode must have been at least 6 months before study initiation. Patients were excluded if they had cancer, were pregnant, had any diseases affecting the immune system, or any known infectious disease.

**Table 1:** General characteristics of controls and patients with VTD.

	Controls (n = 40)	Patients with VTD (n = 50)			P*
		Male (n = 23)	Female (n = 27)	All (n = 50)	
Age (years) (mean, range)	44 (24–52)	41 (28–52)	43 (22–54)	43 (22–54)	NS
Male/female (ratio)	18/22 (0.82)			9:12 (0.75)	NS
Family history of VTD (n, %)	0	17 (73.9)	19 (70.4)	36 (72.0)	
Age at first VTD Episode (mean, range)		22 (16–41)	20 (16–49)	23 (16–49)	NS
Number of VTD events (mean, range)		89 (3, 3–7)	96 (3, 3–8)	185 (3, 3–8)	NS
Type of VTD events					
DVT LL		38 (42.7)	43 (44.8)	81 (43.8)	NS
DVT RL		25 (28.1)	29 (30.2)	56 (27.6)	NS
Bilateral DVT		15 (16.8)	11 (11.5)	26 (14.0)	<0.05
PE		7 (7.9)	9 (9.4)	16 (8.6)	NS
DVT + PE		8 (8.9)	12 (12.5)	20 (10.8)	<0.05
Other		4 (4.5)	4 (4.2)	8 (4.3)	NS

VTD: venous thromboembolic disease; DVT LL: deep venous thrombosis of the left leg; DVT RL: deep venous thrombosis of the right leg; PE: pulmonary embolism; *: male vs. female; NS: not significant.

### Obtaining mononuclear cells (MNCs)

The method was performed as previously described [9]. Blood (100 ml) was diluted 1:1 with phosphate-buffered saline (PBS) (Invitrogen, Grand Island, NY, USA) and overlaid onto an equivalent volume of Ficoll-paque^TM^ Plus (1.077±0.001 g/ml) (GE Healthcare Bio-Sciences AB, Sweden). Cells were centrifuged at 740 g for 30 min at room temperature. MNCs were isolated and washed twice with endothelial basal medium-2 (EBM-2) (Cambrex, Walkersville, MD, USA) supplemented with 3% fetal bovine serum (FBS) (Hyclone, Logan, UT, USA), 10 ng/ml penicillin/streptomycin (Gibco, Grand Island, NY, USA), and 0.25 μg/ml amphotericin B (Gibco) (complete EGM-2 medium). The number of viable nucleated cells was determined in a Neubauer chamber (Marienfeld, Germany) using Turk solution as a diluent (Hycel, México) and trypan blue (Hycel), respectively.

### Culture of ECFCs

Cells were seeded onto six-well tissue culture plates (30 x10^6^ cells/well; Corning, NY, USA) pre-coated with type I rat tail collagen (BD Biosciences, Bedford, MA, USA) and incubated at 37°C with 5% CO_2_ in a humidity-saturated environment. After 24 h of culture, no adherent cells or debris were aspirated and complete EGM-2 medium was added to each well. Medium was changed daily until colonies of ECs (ECCs) appeared. ECCs were counted by visual inspection using an inverted microscope (Olympus CKX41, Tokyo, Japan) under 40x magnification. ECCs were released with TrypLE Express (Invitrogen) from tissue culture plates, suspended in 2 ml of complete EGM-2 media, and plated onto 25-cm^2^ tissue culture flasks (Corning) coated with type 1 rat tail collagen for further passage.

### Culture of human umbilical vein endothelial cells (HUVECs)

The method was performed as previously described [9]. We obtained ten umbilical cords from products at term. HUVECs were isolated by enzymatic activity using Liberase Research Grade (Roche Diagnostics, GmbH, Mannheim, Germany). Cells obtained were first washed with PBS (Invitrogen). The pellets were re-suspended in supplemented EGM-2 medium (Lonza) and supplemented with 10% FBS (Hyclone) and antibiotics. Pellets were then seeded in 10 ml Petri dishes (Corning). Cells in passage 4 were used as a normal positive control for the experiments.

### Semiquantitative RT-PCR analysis for Ephrin-B2 and Eph-B4

Reverse transcription polymerase chain reaction (RT-PCR) was used to analyze the expression of the *Ephrin-B2* and *Eph-B4* genes in ECFC-ECs in patients with VTD and controls. Total ribonucleic acid (RNA) from ECs (1 x10^6^) in passage 4 was isolated with a Trizol purification system (Invitrogen, Carlsbad, CA USA) according to the supplier's instructions. The concentration and purity of RNA were determined by spectrophotometry (Quawell-Q3000) and its integrity was determined in a 2% agarose gel (Roche, Indianapolis, IN, USA). Complementary deoxyribonucleic acid (cDNA) was synthesized using reverse transcriptase (M-MLV) (Invitrogen) with 1000 ng of total RNA.

Primers used for amplification of *Ephrin-B2* and *Eph-B4* genes (IDT, Cambridge, MA, USA) were as follows: *Ephrin-B2* sense 5'-CTC TGT AAC GCC AGA CCA GA-3', anti-sense 5'-ATA GCT GTC GAC CTT CCC CT-3' and *Eph-B4* sense 5'-CCC AGG AGA CAG GGA GCT G-3', anti-sense 5'-GCC CAC CTG GAG GAT GAC TGT G-3'. The constitutive gene ribosomal protein S18 (*PRS18*) was used as control: sense 5'-AAT CCA AAG TAC CAG ATC CGC CCA-3', anti-sense 5'-TTT CTT GGA GGT CAC CCA-3'. PCR conditions were as follows: one cycle at 95°C for 5 min, then 35 cycles of denaturation at 95°C for 30 sec, aligning at 60°C (*PRS18*), 58°C (*Ephrin-B2*), and 63°C (*Eph-B4*) for 60 sec, and extension at 72°C for 60 sec followed by a final extension at 72°C for 10 min. Eight μl of each PCR product was loaded with SYBR Gold (Invitrogen) in a 2% agarose gel.

Densitometry analysis was performed using an image analyzer (BioRad). As positive control for gene expression of *Eph-B4* and *Ephrin-B2*, primary cultures of HUVECs and pulmonary artery endothelial cells (PAEC) of normal humans (ATCC-used PCS-100-022) were used. In both cases, cells used were at passage 4.

### Confocal microscopy analysis

ECFC-ECs from VTD patients and controls as well as HUVECs at passage 4, were grown with type I rat tail collagen-coverslips, in 6-well plates at 80% of confluence. Cells were rinsed twice in PBS, fixed in 4% paraformaldehyde in complete medium for 5 min, then fixed in 4% paraformaldehyde in PBS, and permeabilized in 0.1% Tween 20 (Biorad) for 1 h. After washing three times with PBS, coverslips were incubated at 4°C overnight with a rabbit polyclonal anti-Ephrin2 and anti-Eph-4 IgG antibodies (Santa Cruz Biotechnology, USA), and with a rabbit monoclonal anti-gamma H2A.X (phospho S140) antibody [γH2AX] (Abcam, Eugene USA). After washing with PBS, cells were stained with 0.4% DAPI and incubated with a goat anti-rabbit Alexa Fluor 488 antibody (ThermoFisher Scientific, IL, USA), at room temperature during 40 min. Then, cells were washed with PBS and mounted in an aqueous media VECTASHIELD (Vector Laboratories, Inc. Burlingame, CA.). Cell morphology and intracellular localization of proteins were analyzed by a confocal microscope (Nikon Ti Eclipse, USA).

### Growth kinetics and estimate of replicative capacity of ECFC-ECs

At passage 2, 1 x10^4^ ECFC-ECs were plated in six-well plates with 2 ml EGM-2. Subsequently, when 80% cell confluence was observed, cells were harvested with TrypLE Express and counted using trypan blue. At each subsequent passage, cells were counted for calculation of a growth kinetic curve, population-doubling times (PDTs), and cumulative population-doubling levels (CPDLs). The number of PDs occurring between passages was calculated according to the following equation:

PD=(Ln(numberofcellscounted/numberofcellsatthebeginningoftheassay)/Ln2).

The sum of all previous PDs determined the CPDL at each passage. PDT was obtained by means of the time interval between cell seeding and cell harvest divided by the number of PDs for each passage [14].

### FACS analysis

Measurement of intracellular H_2_O_2_ levels in 5 x10^4^ ECFC-ECs in suspension at passage 4 was performed after loading with 2′,7′-dichlorodihydrofluorescein diacetate (DCF-DA, 20 μM; MitoSciences, Cambridge, MA, USA) for 30 min at 37°C in the dark, washed with PBS, and detached with TrypLE Express. After centrifugation, cell pellets were suspended in 200 μl PBS. Formation of ROS was detected by the signal obtained from the fluorescent reaction products 2’,7’-dichlorofluorescin (DCF) and ethidium. Cells were washed and analyzed by means of fluorescence-activated cell sorting (FACS) (FACS Calibur, Becton Dickinson, USA). Results were analyzed using the FlowJo Software v.7.6.5 (Ashland, OR, USA). The absolute values of mean fluorescence intensity (MFI) were expressed as percentages and the result obtained from controls was considered as the 100% reference value. For determination of apoptosis, ECFC-EC in passage 4 were detached with TrypLE Express for 5 min at 37°C, washed in PBS, and suspended in binding buffer (BD Biosciences). After centrifugation, cell pellet was suspended in 50 μl binding buffer and incubated for 15 min at room temperature with 2 μl annexin–fluorescein isothiocyanate (FITC; BD Biosciences) and 2 μl propidium iodide (PI; BD Biosciences). Binding buffer (300 μl) was added and cells were washed twice and analyzed by means of FACS. We analyzed the results using FlowJo Software v.7.6.5.

To evaluate the expression of γH2AX in ECFC-ECs from controls and VTD patients at passage 4, cells were grown with type I rat tail collagen in 6-well plates at 80% of confluence. Cells were rinsed twice in PBS, fixed in 4% paraformaldehyde in PBS for 20 min, and permeabilized with 0.1% Triton 100 X (Sigma Aldrich USA), in PBS for 20 min at 4°C. Then, cells were blocked in a 1% BSA solution in PBS and incubated with a murine anti-human anti-H2AX (pS139) PE-conjugated antibody (BD Biosciences). Labeled cells were analyzed in a FACSVerse (BD Biosciences), and the data obtained were evaluated with a Flowjo software (Version 10.3).

### Senescence-associated β-galactosidase activity assay

Senescence-associated β-galactosidase (SA-β-gal) activity was measured as described previously. Briefly, adherent EPC at passages 4 and 10 were washed in PBS, fixed for 60 min at room temperature in 2% paraformaldehyde, washed and incubated for 24 h at 37°C without CO_2_ and with a fresh SA-β-gal stain solution: 1 mg/ml 5-bromo-4-chloro-3-indyl-β-D-galactopylanoside (X-gal; Promega, Madison, WI, USA), 5 mmol/l potassium ferrocyanide, and 2 mmol/l MgCl_2_. After two additional washes with PBS, stained cells (blue) were observed with phase contrast microscopy. Cell counting was done in 100 different fields.

### Proliferation of ECFC-ECs

We prepared a mixture of plasmas from four controls and four patients with VTD (two males and two females). Conditioned mediums (supernatants) from ECFC-ECs of four controls and four VTD patients were collected at passage 4 after 24 h of culture (80% of confluence). ECFC-ECs (2 x10^3^ cell/well) from patients and controls at passage 4 were plated in triplicate in 24-well plates under three different conditions: a) EGM-2 medium supplemented with 10% FBS; b) EGM-2 medium supplemented with 10% plasma from patients with VTD; and c) EGM-2 medium supplemented with 10% of control plasma. Treatments with the supernatants were done as follows: a) EGM-2 medium supplemented with 10% FBS; b) EBM-2 medium conditioned 24 h with ECFC-ECs from patients with VTD and supplemented with 10% FBS; and c) EBM-2 supernatants 24 h with EPC from patients with VTD and supplemented with 10% FBS. The fold-increase (FI) population was determined by dividing the number of living cells on day 7 by the living cells at day zero. Medium changes were performed every 24 h for 7 days of culture and cells were harvested with TrypLE Express. Live cells were counted and then evaluated with trypan blue.

### Statistical analysis

Results are expressed as mean ± standard deviation. T-test and One-way analysis of variance test were used to analyze the results; P ≤0.05 was considered statistically significant. Data were analyzed with SigmaStat statistical software (v.3.5, Sigma Stat, San Jose, CA, USA).

### Ethical considerations

The study protocol was approved by the National Ethics Committee of the Instituto Mexicano del Seguro Social (authorization number IMSS-R-2014-785-030 and IMSS-R-2015-785-091). All participants were informed about study procedures and informed, written consent was obtained from each individual before blood samples were obtained. This study fulfilled the principles of the Declaration of Helsinki. Human umbilical cord blood was always obtained according to institutional guidelines.

## Results

### Increased number of ECFCs from VTD patients

**Fig 1:**
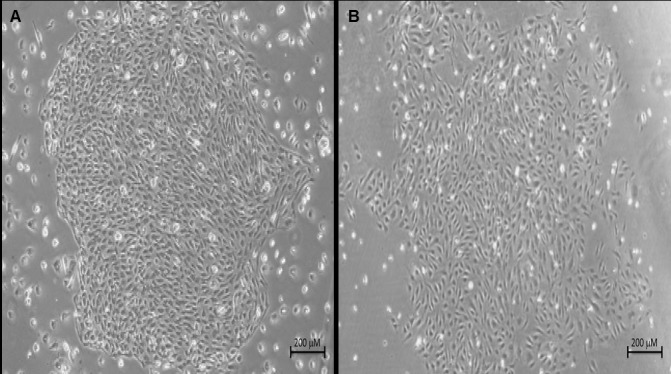
Identification of ECFCs obtained from peripheral blood from controls and patients with VTD. Representative photomicrograph (40X magnification) of colonies formed by ECFCs in cultures of MNCs obtained from peripheral blood after 14 days in culture. (A) controls and (B) patients with history of VTD.

**Fig 2:**
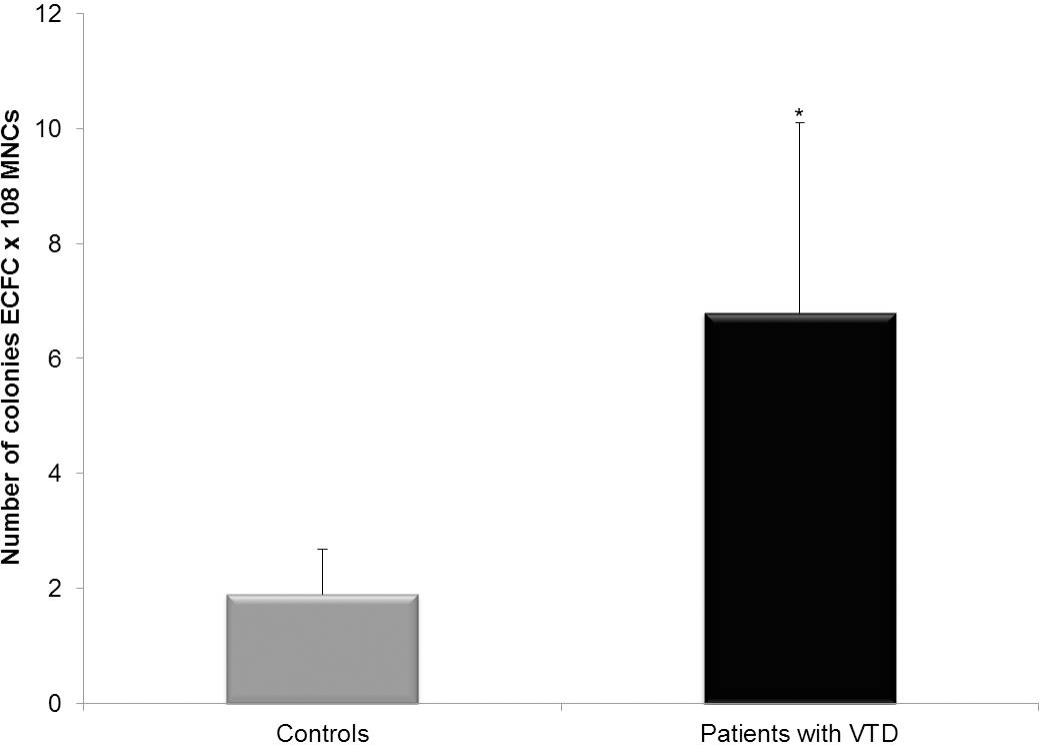
Frequency of ECFCs obtained from peripheral blood of controls and patients with VTD. This figure shows the number of ECFC colonies obtained from peripheral blood of controls and patients with VTD per 10^8^ MNCs seeded in culture plates. Results are expressed as mean ± standard deviation of 16 independent samples in each case. *P = 0.001.

ECCs isolated from the MNC fraction of blood samples from 40 controls (Fig 1A) and 50 VTD patients (Fig 1B) had similar morphological characteristics and the typical cobblestone morphology. In addition, number of ECCs from VTD patients in culture per 100 x10^6^ MNCs was higher than in controls (6.8±3.3 vs. 1.9±0.8, P ≤0.001) (Fig 2). These results suggest that ECCs from VTD patients may have abnormal cell proliferation and angiogenic potential that may affect endothelial remodeling.

### Impaired expression of Eph-B4/ephrinB2 in ECFC-ECs from patients with VTD

**Fig 3:**
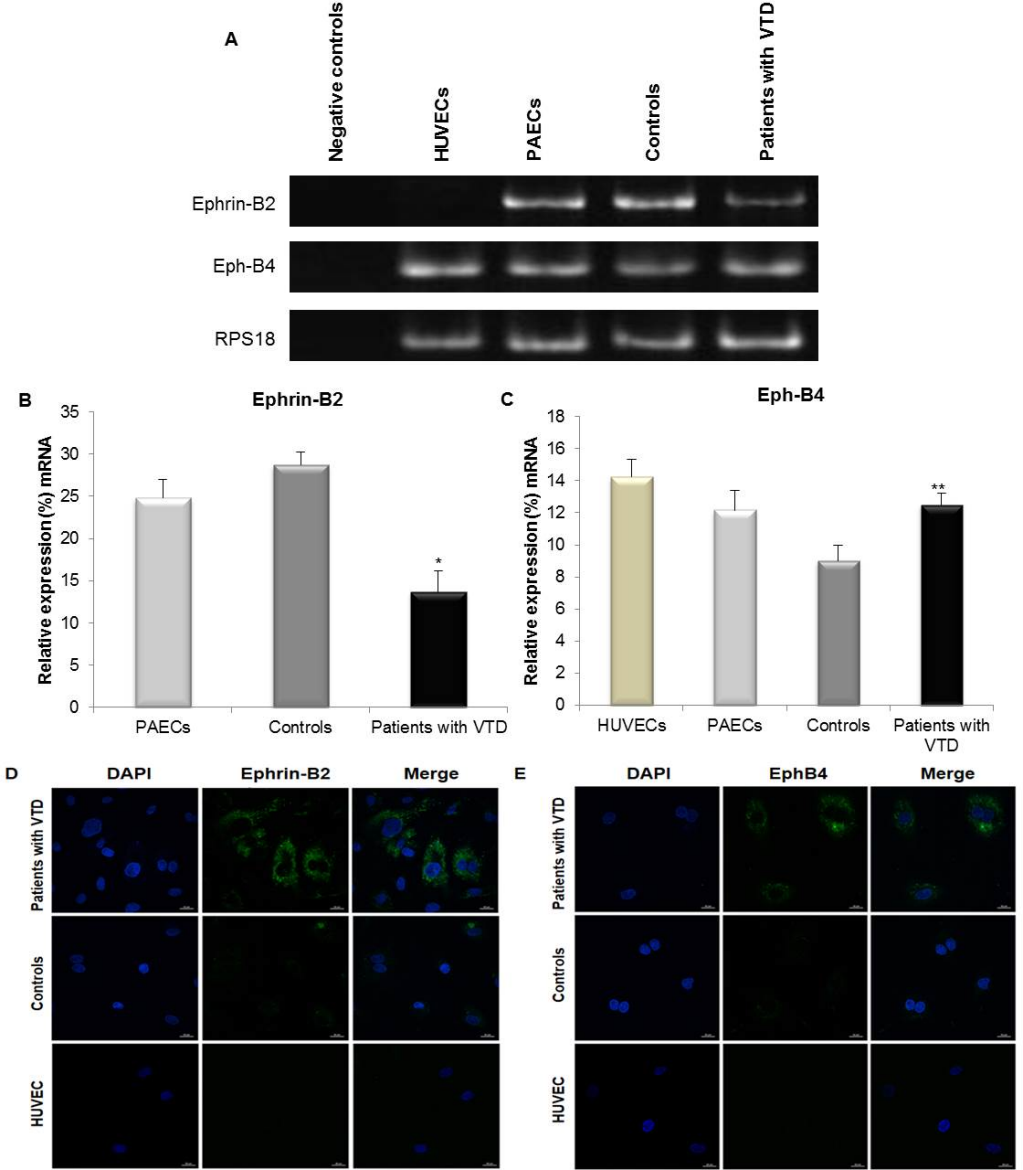
Relative expression of ephrin-B2 and Eph-B4 genes in ECFC-ECs from peripheral blood of controls and patients with VTD. **(**A) mRNA expression of ephrin-B2 (639 bp), Eph-B4 (251 bp) and RPS18 (240 bp) as housekeeping gene in peripheral blood samples from controls and from patients with VTD. (B) Densitometric analysis of ephrin-B2 and (C) Eph-B4 evaluated in positive controls (HUVECs and PAECs) in ECFC-ECs of controls and patients with VTD. Results are expressed as mean ± standard deviation of three independent experiments in passage 4. *P = 0.017, **P = 0.01. Expression of the proteins Ephrin-B2 (D) and Eph-B4 (E), was evaluated by confocal inmunofluorescence microscopy.

During embryonic development, regulation of the vascular network generation depends on the receptor EphB4 and its ligand ephrin-B2, both markers for venous and arterial beds, respectively. However, they remain important during adult life for tissue regeneration as well as for the pathophysiology of some diseases. We hypothesized that in ECFC-ECs from patients with VTD, the expression of this complex was different as compared with controls. We evaluated ECFC-ECs isolated from VTD patients and controls for their expression of EphB4 and ephrin-B2 by RT-PCR. PAECs were used as a positive control for ephrin-B2 and HUVECs were used as a positive control for EphB4 (Fig 3A). Densitometry analysis shown that expression of specific transcripts of Eph-B4 was increased (12.5±0.6) as compared with controls (9.5±0.5). Interestingly, ephrin-B2 was reduced in ECFC-ECs from patients with VTD (13.7±2.5) vs. controls (28.7±1.5) P = 0.017 (Fig 3B and 3C, respectively). However, when we analyzed the presence of the protein by immunofluorescence we detected and increase in the expression of both, EphB4 and Ephrin-B2 as compared with their normal counterparts (Fig 3D and 3E, respectively). These data suggest the presence of abnormal mechanisms of proliferation and revascularization or ECFC-ECs obtained from VTD patients.

### Reduced proliferation in ECFC-ECs from patients with VTD

**Fig 4:**
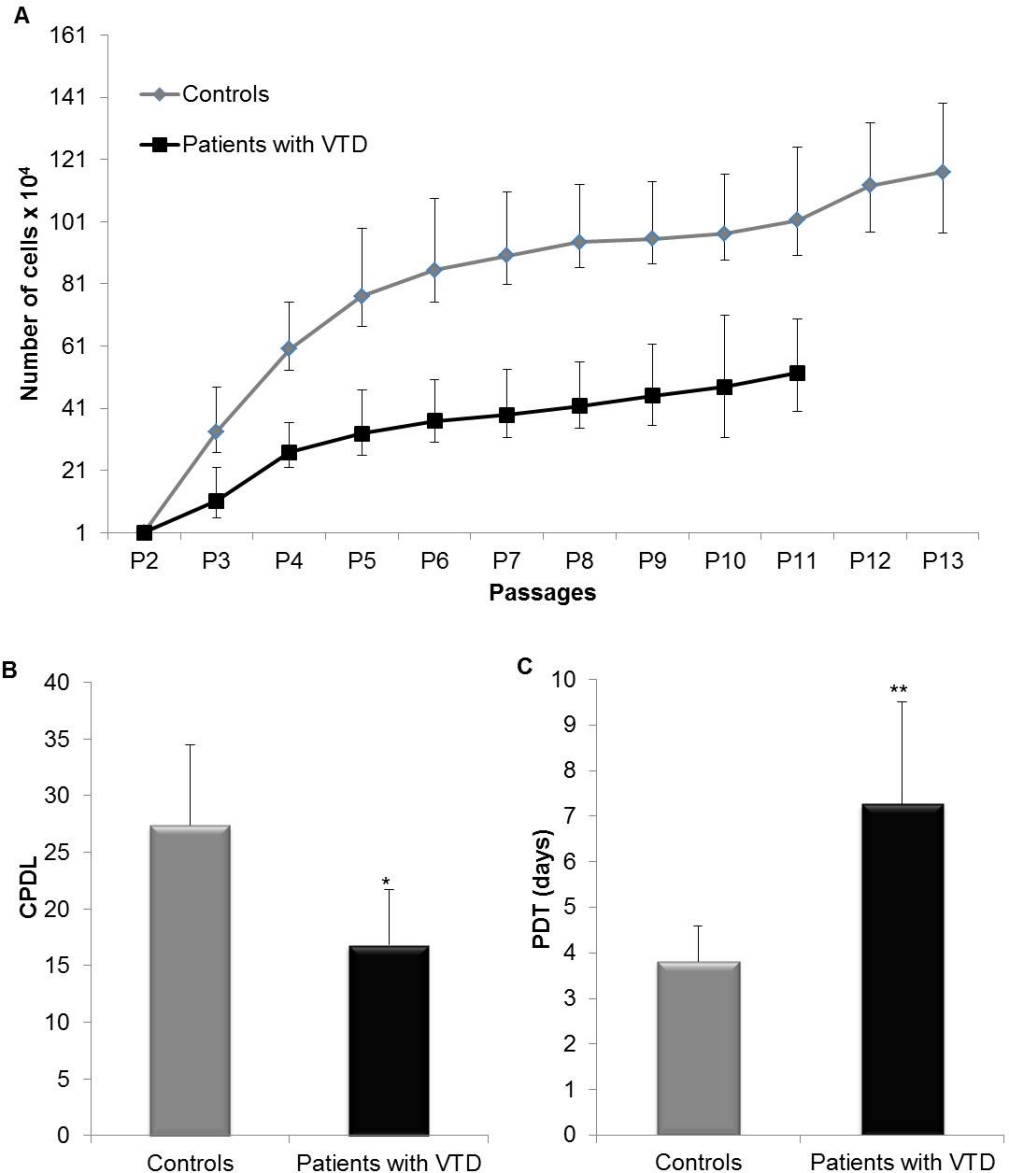
Proliferative potential of ECFC-ECs from peripheral blood of controls and patients with VTD. This figure shows the results from ECFC-ECs obtained from VTD patients and controls regarding the following: (A) Proliferation of ECFC-ECs. Each point on the graph indicates the accumulated cell number along different reseedings. (B) Cumulative population doubling (CPD) levels. (C) Population doubling times (PDT). Results are expressed as mean ± standard deviation of five independent experiments. *P = 0.032 for CPDs and **P = 0.017 for PDTs.

ECFC-ECs from adult peripheral blood have good proliferation potential but limited as compared with ECFC-ECs from human umbilical cord blood. To investigate the potential expansion of ECFC-ECs from patients with VTD and controls, we analyzed the cell growth kinetics at different passages by generating growth curves. We found that ECFC-ECs from control at earlier (4) and late (12) passages have superior growth kinetics and reached higher densities at confluence than cells from patients with VTD (39% reduced proliferation rate) (Fig 4). To evaluate and quantitate the proliferation rate of ECFC-ECs from VTD patients vs. controls, we analyzed the profiles of population doubling times (PDT) as well as the cumulative population doublings (CPD) at passage 11 in the culture. We observed that ECFC-EC from controls have 27.37±7.07 PDT vs. 16.81±4.85 PDT in ECFC-ECs from VTD patients (P = 0.032) (Fig 4B). We also observed that PDT required by ECFC-ECs from controls was 3.80±0.79 days, whereas it was 7.26±2.24 days for ECFC-ECs from patients with VTD (P = 0.017) (Fig 4C). This finding strongly suggests that there are significant deficiencies in the proliferative response of ECFC-ECs from patients with VTD.

### ECFC-ECs from patients with VTD exhibit high rates of ROS

**Fig 5:**
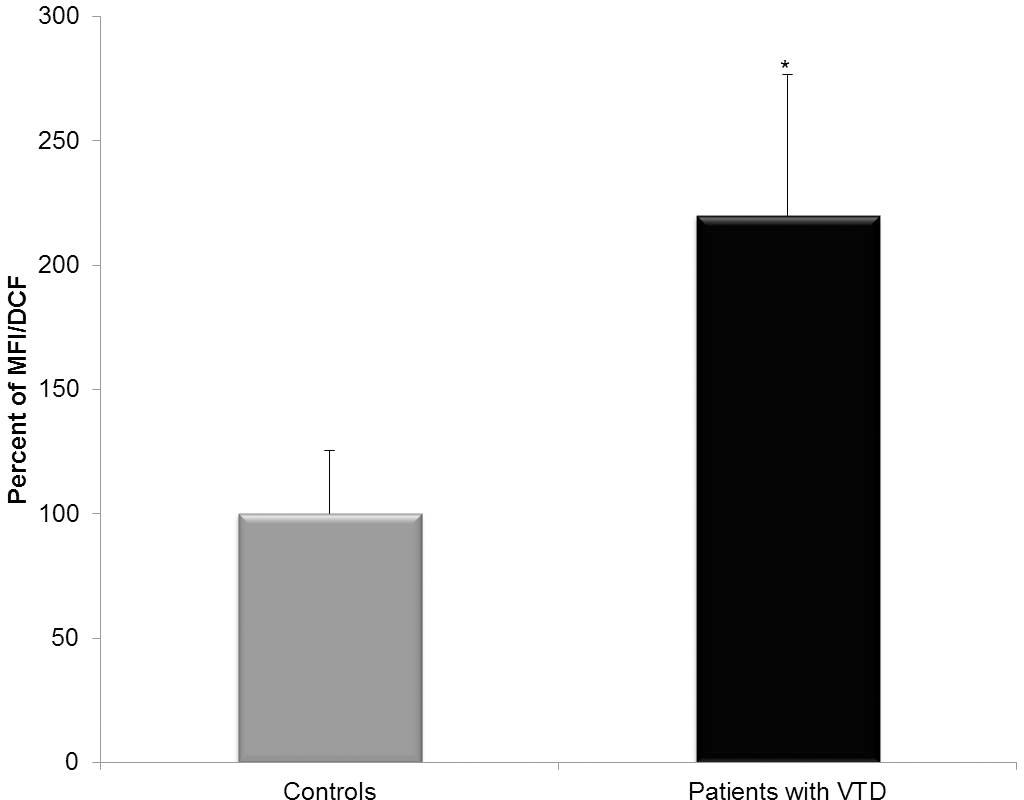
Expression of ROS in ECFC-ECs obtained from blood of controls and patients with VTD. Mean fluorescence intensity (MFI) emitted by 2’,7’-dichlorofluorescein (DCF) in ECFC-ECs from peripheral blood samples of controls and patients with VTD in passage 4. MFI was expressed as percentage being 100% the value obtained by ECFC-ECs from controls. The results are expressed as mean ± standard deviation of three independent experiments *P = 0.012.

In a previous study, we reported that ECFC-ECs from patients with VTD have significant abnormalities at the mitochondrial membrane level [9] and a reduced proliferation rate, which suggested a higher presence of ROS (specifically H_2_O_2_). We then analyzed ROS levels in ECFC-ECs from patients with VTD and controls with DCF-DA by means of fluorescence-activated cell sorting (FACS). We found a significant increase of intracellular H2DCF oxidation in ECFC-ECs from patients with VTD (1,775.67±459.08 MFI) as compared with ECFC-ECs from controls (807.0±205.75 MFI) (p = 0.012) (Fig 5).

### Apoptosis in ECFC-ECs from patients with VTD

**Fig 6:**
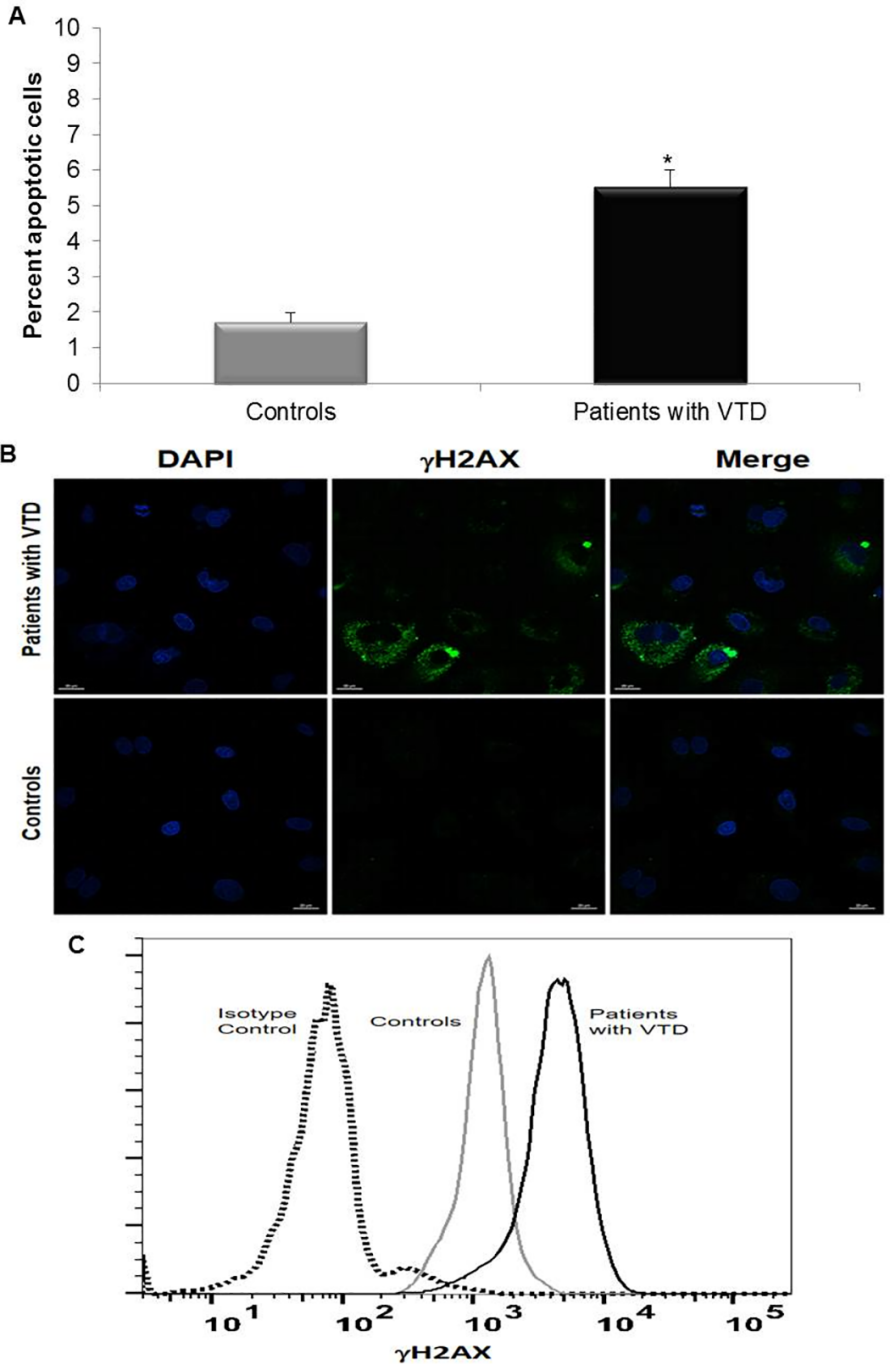
Apoptosis in ECFC-ECs obtained from peripheral blood of controls and patients with VTD. The figure shows the percentage of ECFC-EC annexin V-FITC positive (A) and the presence of DNA damage evaluated by the expression of γH2AX histone by immunofluorescence (B) as well as flow cytometry analysis (C), in passage 4. Results are expressed as mean ± standard deviation of three independent experiments. *P = 0.002 for annexin expression.

Once we demonstrated that ECFC-ECs from patients with VTD exhibited increased intracellular ROS levels, we analyzed the degree of apoptosis by means of the expression of annexin V-FITC. A significant increase in apoptosis in ECFC-ECs from VTD patients (5.52±0.46%) was observed vs. expression of the controls (1.73±0.25%) (Fig 6A). These results clearly demonstrated that ECFC-ECs from patients with VTD are more sensitive to ROS-induced apoptosis. We also analyzed the DNA damage by means of the expression of the associated phosphorylated histone H2AX (γH2AX) by immunofluorescence (Fig 6B), and flow cytometry analysis (Fig 6C). Our results show an increase in the level of DNA damage in ECFC-ECs from patients with VTD as compared with controls.

### Senescence in ECFC-ECs from patients with VTD

**Fig 7:**
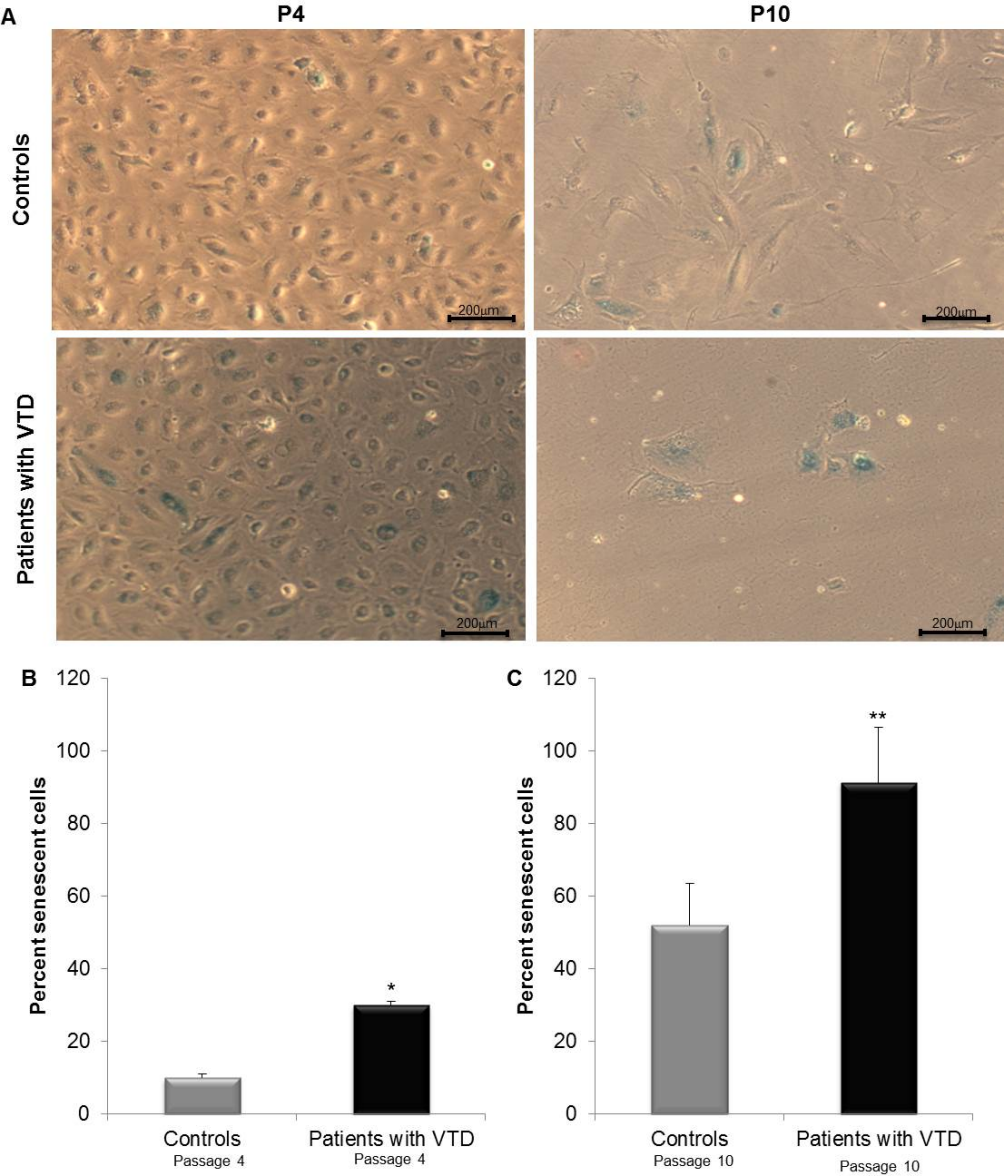
Senescence in ECFC-ECs obtained from peripheral blood of controls and patients with VTD. SA-β-gal activity analysis was used as a marker of cell senescence. (A) Representative images from controls and patients with VTD-ECFCs at passage 4 (P4) and 10 (P10) (upper panel, magnification X100; scale bar, 200 μm). (B) Percentage of senescent cells in patients with VTD-ECFCs was determined with the number of cells expressing SA-β-gal (blue cells) related to the total number of cells/field from patients with VTD (n = 3) or controls (n = 3). Graph (lower panel) represents mean ± SEM of five independent samples. Experiments were done in triplicate. *P = 0.001 (patients with VTD vs. controls at passage 4); **P = 0.023 (patients with VTD vs. controls at passage 10).

Because an impaired expression of Ephrin-B2 and eph-4 genes as well as reduced proliferative capacity and ROS in ECFC-ECs from VTD patients were found as compared with controls, we attempted to determine whether these abnormalities were likely associated with senescence because morphological changes and appearance of enlarged flattened cells in ECFC-ECs from VTD patients were observed. Therefore, we investigated the rate of senescence using SA-B-gal staining. ECFC-ECs from patients with VTD were highly senescent (30±1.05%) at passage 4 as compared with controls (10±0.98%) (Fig 7A and 7B) (p = 0.001). Interestingly, at passage 10, the senescence increased to 52.09±11.44% and 91.3±15.07% in ECFC-ECs from controls and patients with VTD, respectively. These results strongly suggest that, in ECFC-ECs from patients with VTD, addition of the above-mentioned abnormalities may reduce their proliferative capacity and may induce increased senescence.

### Effect of plasma and supernatants on the proliferation of ECFC-ECs in patients with VTD

**Fig 8:**
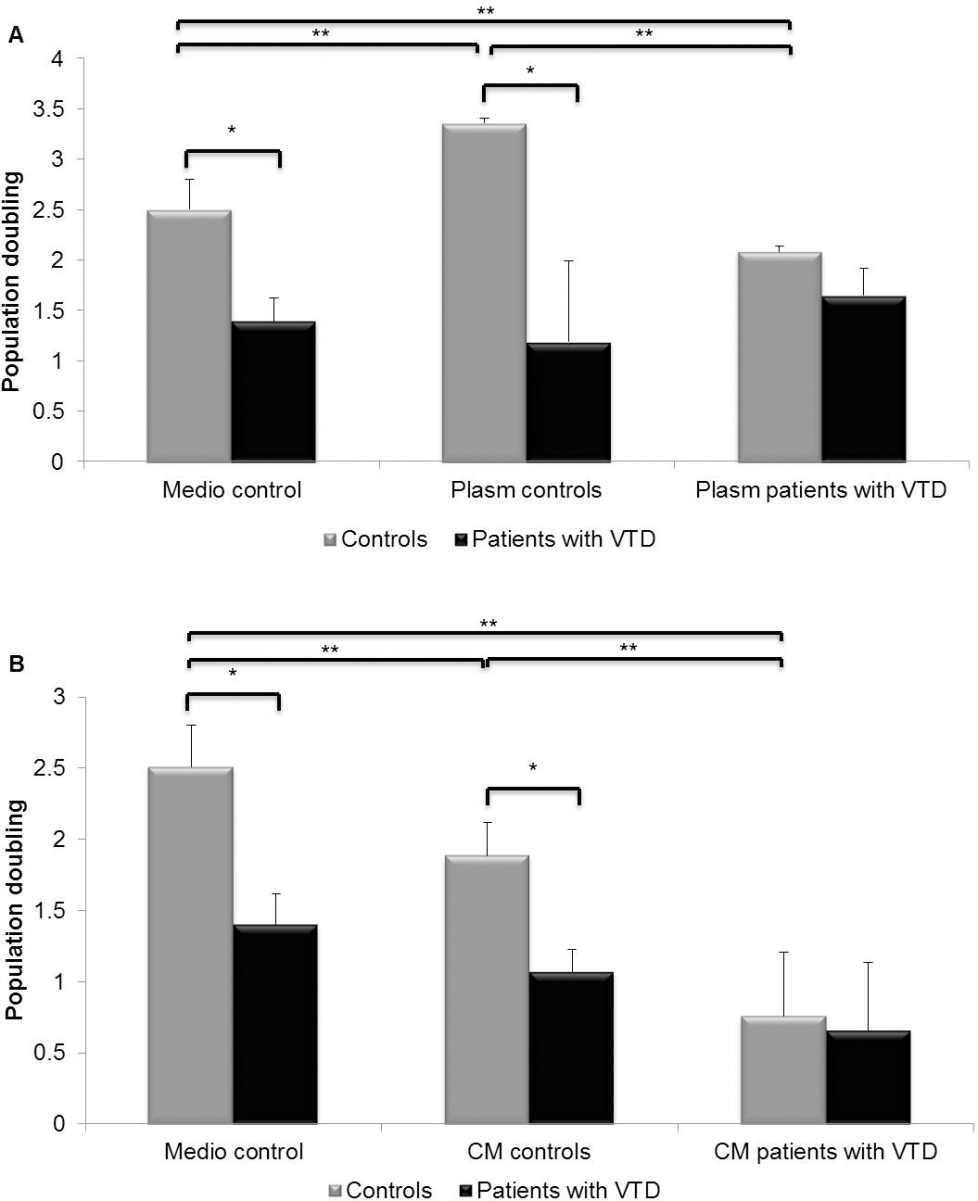
Effect of plasma and supernatants on the proliferative potential of ECFC-ECs obtained from peripheral blood of controls and patients with VTD. (A) The figure shows the number of ECFC-ECs in controls and patients with VTD at passage 4 after stimulation with plasma obtained from controls and patients with VTD. (B) Results of the 24-h incubation of supernatants and ECFC-ECs from VTD patients and controls. Results are expressed as mean ± standard deviation of three independent experiments, *P ≤0.05, **P ≤0.05.

In order to investigate the likely role of the microvascular environment on the proliferative potential of ECFC-ECs obtained from patients with VTD and controls, we substituted the bovine fetal serum used in the medium (control medium) with previously collected plasma from patients with VTD and controls. Fig 8A shows that the proliferative potential of ECFC-ECs obtained from patients with VTD was lower when incubated with control medium [1.4±0.22 doubling population (DP)], plasma from control subjects (1.18±0.31 DP), or with plasma from VTD patients (1.65±0.27 DP). In contrast, ECFC-ECs obtained from control individuals showed a higher proliferative capacity under the same three previously described culture conditions: control medium (2.5±0.44 DP), plasma obtained from control individuals (3.36±0.03 DP), and plasma obtained from patients with VTD (2.080±0.25 DP). However, it should be emphasized that when these last cells were incubated with plasma from control individuals, the proliferation rate was significantly higher as compared with the results obtained with control medium and plasma obtained from patients with VTD.

In another series of experiments, we used control medium or supernatants of the ECFC-ECs cultures (incubated with conditioned medium) obtained from patients with VTD or control subjects. Fig 8B shows that the proliferative potential of ECFC-ECs from VTD patients incubated with control medium (1.39±0.22 DP), supernatants of ECFC-ECs obtained from control individuals (1.06±0.16 DP), or supernatants obtained from patients with VTD (0.65±0.48) was significantly lower as compared with the proliferative potential of the ECFC-ECs obtained from control individuals when incubated with control medium (2.50±0.29 DP), supernatants of ECFC-ECs from control individuals (1.88±0.23 DP) or supernatants of ECFC-ECs obtained from patients with VTD (0.760±0.45 DP).

## Discussion

VTD is currently considered a public health problem although its exact incidence is unknown. It is calculated that each year 10 million people are affected by a new episode of VTD worldwide. As a consequence, VTD is considered a major contributor to the global disease burden. This is one of the major reasons why it is important to investigate the unknown risk factors associated with this disease as well as to delve more deeply into its pathophysiological mechanisms in order to reduce the high rates of morbidity and associated mortality in the near future [1,15].

EPCs were described in 1997 [16] and a body of studies immediately began in order to identify their contribution to several vascular diseases such as arterial hypertension [17], atherothrombotic disease, cerebral ischemia [18], and diabetes [19]. It has been described that ECFCs are sensitive to oxidative stress (a phenomenon described in acute myocardial infarction) [20] and splanchnic vein thrombosis [5]. However, in general, their contribution to the pathogenesis of other vascular abnormalities has been poorly analyzed. Because there are no reports describing the association of ECFC abnormalities and oxidative stress, we attempted to gain information in patients with recurrent, chronic VTD.

Our study focused on the evaluation of the effect of oxidative stress on the potential proliferation of ECFC-ECs obtained from the peripheral blood of patients with VTD. We aimed to understand whether such a mechanism could be related to venous thrombotic events in patients experiencing recurrent episodes in whom an endothelial dysfunction may be present, despite the use of anticoagulant therapy such as vitamin K antagonists or rivaroxaban. We did not find significant differences in terms of the size and shape of ECCs obtained from controls and patients with VTD. Interestingly, in patients with VTD, we found an increase of circulating ECCs, suggesting a mechanism of response to chemotactic signals that could induce a dysfunctional endothelium in order to replace ECs and to promote revascularization of the damaged vascular network [21]. This is the case of patients with acute myocardial infarction in whom there is an increase of EPCs that apparently favors the vascular repair process [4].

Recent evidence found an increased frequency of EPCs in the peripheral blood of patients with coronary artery disease and perhaps may be considered an indicator of risk for suffering a new thrombotic event [22]. It is suggested that intracellular ROS are a determining factor in the mobilization of EPCs resembling a phenomenon occurring with hematopoietic stem cells (HSC) and hematopoietic progenitor cells (HPC) as they are released from the bone marrow [23]. In this regard, expression of transcripts Ephrin-B2 and Eph-B4 in EPCs plays an important role in vascular formation during embryonic development and postnatal revascularization processes, which coordinate venous or arterial traffic of ECs in case of vascular repair. We found increased transcript expression of Eph-B4 and a low transcription expression for Ephrin-B2 in ECFC-ECs from patients with VTD. Both findings were consistent with previous data demonstrating an increased Eph-B4 transcript expression in EPCs from mouse and human. This plays an important role in the remodeling of venous endothelium [24] as well as in vein formation during embryonic and postnatal development, respectively [25]. In this study, we found an increased expression of Eph-B4 and Ephrin-B2 proteins in ECFC-ECs from patients with VTD and we believe that this phenomenon could be associated with major abnormalities occurring in these cells.

The increased frequency of circulating ECFC-ECs and the high expression of Eph-B4 observed in patients with VTD suggest they have a high proliferative capacity, which may be useful to induce an efficient revascularization process. However, when we evaluated their proliferation potential, they showed a reduced proliferation rate of 50% at 4 months of time (times at which we eliminate the cultures). This fact shows a reduction of 15 duplications and 4 days in order to double the cell population. Our data agree with previous reports that indicated a low rate of EPC proliferation in patients with hypercholesterolemia, intrauterine growth restriction, and heart disease [26,27]. In our study, we attempted to find changes in the angiogenic function of CPEs secondary to a challenging environment capable of inducing oxidative stress [28]. Mitochondria are involved due to having several important metabolic functions such as calcium regulation and activation of cell death, being the most important inductors of ROS. As a consequence, abnormal mitochondrial function may accelerate some processes like inflammation, cell senescence, and apoptosis. Recent evidence indicates that mitochondrial dysfunction contributes significantly to the pathogenesis of cardiovascular disease, an entity characterized by damaged DNA [29].

We found morphological changes in mitochondria and an increased pro-inflammatory cytokine profile (IL-6, IFN-γ, and TNF-α) in the supernatants of cultures of ECFC-ECs from VTD patients [9]. Our results also showed increased ROS levels produced by ECCF-ECs of VTD patients, specifically hydroxyl radicals and hydrogen peroxide, a fact that strongly suggests that the low proliferative rate observed in these cells may be closely related to their dysfunctional status. This low proliferative rate may be related to the overproduction and chronic exposure to oxidative stress either generated by the resident vessel cells or during the migration process from circulation to damaged vessel sites that require remodeling. Although the mechanisms responsible for the dysfunction of EPCs are complex and still not fully understood, we know that oxidative stress plays a key role in EPC dysfunction of some cardiovascular diseases such as hypertension, hyperlipidemia and stroke. Therefore, we believe that this oxidative stress may be a significant contributor to the dysfunctional status observed in ECFC-ECs from patients with recurrent VTD [30,31].

Some studies suggest that EPCs are more resistant to oxidative stress because of their higher expression of antioxidant enzymes that protect themselves, thus allowing a more appropriate repair of blood vessels damaged in the hyperoxidative environment of ischemic tissues [32,33]. Some events associated with an increased oxidative stress may lead to mobilization of dysfunctional EPCs [34]. This suggests the presence of abnormal proliferative mechanisms such as cyclin D1 synthesis or activation of cyclin-dependent kinase 4 (CDK4) [35] and lower cell survival mediated through regulation of anti-apoptotic components such as BCL-2 [36]. This may lead EPCs from patients with VTD to an accelerated apoptotic or cell senescence condition, two mechanisms capable of reducing the ability of EPCs to properly work in the revascularization procedures at endothelial level.

Although there is a significant difference in the percentage of cells in apoptosis between ECFC-ECs from patients with VTD and controls, in both cases it is relatively low, considering that apoptosis has a major role in reducing the proliferation rate of EPCs. *In vitro* tests have shown that high concentrations of hydrogen peroxide are necessary to induce >10% of apoptosis in EPCs [37]. Similarly, it was reported that EPCs derived from cord blood are more resistant to cell death compared with HUVECs, suggesting that EPCs are less susceptible to apoptosis [14]. Interestingly, our data show increased levels of DNA damage, an event that could be related with cellular senescence. However, it is important to underline that we also found in cytoplasm expression of the γH2XA enzyme in ECFC-ECs from VTD patients, a fact that may suggest that the presence of extra-nuclear DNA is the result of abnormal activation of signal pathways related to cell death as previously described in other cell types [38]. In addition, it has been reported that the exogenous presence of histones in CFCE can inhibit proliferation and angiogenic function inducing apoptosis or pyroptosis, through the formation of pores or use existing channels through the depolarization of the cellular membrane and the influx of calcium [39, 40]. This leads us to assume that in CFCE-EC from patients with VTD, the intrinsic presence of γH2XA enzyme is directly related to events of angiogenesis and active vasculogenesis such as ischemic tissue [41]. However this interesting issue remains to be investigated, because in our previous report we identified alterations of mitochondrial membrane in ECFC-EC from VTD patients [9].

Several conditions associated with an increased oxidative stress, e.g., hyperhomocysteinemia and organ ischemia, have been linked to EPC dysfunction, particularly through the induction of senescence in adults and neonates [42, 43]. In our study, senescence was related to the activity of β-galactosidase in ECFC-ECs from patients with VTD at passage 4 as also occurs in other *in vitro* studies. This fact strongly suggests that oxidative stress induces an early entry to cellular senescence because it may occur in in EPCs from preterm neonates [42,44] as well as from adults with coronary artery disease [45]. In our study, in patients with VTD, the observed senescence could be induced by oxidative stress and not exclusively by telomere shortening as occurs in replicative senescence [46].

Studies addressing cellular senescence of EPCs have not been carried out as in our study, except in the study by Vassallo et al. who observed 85% increase in EPC senescence in culture up to passage 10. Based on this finding we believe that ECFC-ECs from patients with VTD have normal capabilities leading to an early senescence, perhaps through an increase of p16^INK4a^ inhibitor, which would induce cell arrest in the G0/G1 phases of the cell cycle [38,47,48].

We attempted to determine if the low proliferation potential, the increase of ROS, and the cellular senescence observed in ECFC-ECs from patients with VTD were likely caused by pro-inflammatory cytokines present in the plasma or supernatants of cultures from ECFC-ECs as recently demonstrated [9]. We found that ECFC-ECs from patients with VTD exhibit a low proliferative response to angiogenic stimuli of vascular endothelial growth factor (VEGF) in normal plasma and in the supernatants of ECFC-ECs obtained from controls. Also, proliferation of ECFC-ECs obtained from controls was significantly decreased after being incubated with plasma or supernatants from ECFC-ECs obtained from patients with VTD. Similar results were found by other groups describing that in plasma and supernatants of EPCs from patients with pulmonary hypertension and heart disease, there are factors inducing an anti-angiogenic effect that accelerate the onset of early senescence in EPCs through increased oxidative stress [49–51]. These data strongly suggest that dysfunction of ECFC-ECs observed in patients with VTD may be caused by intrinsic factors that induce the activation of one or several senescence pathways related to oxidative stress that may affect the normal proliferative response of progenitor cells to external stimuli and, consequently, predisposing to endothelial dysfunction and subsequently, to new thrombotic events in patients with VTD.

This is the first study addressing the likely effects of oxidative stress on EPCs from patients with a history of VTD after 4 months of culture. Future studies are needed to define the role of ROS and signaling pathways on stress-induced CPE senescence before being considered as an option to promote endothelial repair and regeneration in patients with thrombotic diseases.

## Conclusions

Our study shows that dysfunction of ECFC-ECs obtained from patients with VTD is associated with increased oxidative stress and cellular senescence. Moreover, our results strongly suggest that patients with a history of VTD may be at risk of new thrombotic events due to a low proliferative potential of their CPEs, a fact that secondarily may be associated with a defective response to abnormal changes in the vascular microenvironment, perhaps leading to subsequent thrombotic events.
